# Alcohol intoxication impedes the recognition of traumatic brain injury in the prehospital setting and may worsen 6-month outcome

**DOI:** 10.1186/cc12259

**Published:** 2013-03-19

**Authors:** R Raj, J Siironen, R Kivisaari, M Kuisma, M Skrifvars

**Affiliations:** 1 Helsinki University Central Hospital, Helsinki, Finland

## Introduction

Transport directly to a neurosurgical trauma center has shown to reduce mortality in patients with traumatic brain injury (TBI). We hypothesized that alcohol intoxication may impede prehospital recognition of TBI, resulting in transportation to a non-neurosurgical hospital.

## Methods

A retrospective analysis of TBI patients admitted to a designated neurosurgical trauma center's ICU in 2009/10 and primarily treated by the Emergency Medical Service (EMS). Patients were divided into two categories based either direct or indirect trauma transfer by the EMS. Directly transferred patients are directly transported to the neurosurgical trauma center from the injury scene while indirectly transported patients are initially transported to another non-neurosurgical hospital before re-transfer to the trauma center. Data from patient journals and EMS forms were extracted. The blood alcohol level (BAL) was measured by the EMS using an alcohol breath-test. Logistic regression modeling was used to identify variables present at scene associated with transport destination.

## Results

Totally 470 patients met the inclusion criteria; 60% were transported directly and 40% indirectly. In the direct group 15% of patients had a positive BAL, compared with 26% for those indirectly transported. In the logistic regression model, factors associated with direct transport were: BAL ≥2.3‰ (OR: 0.06, CI: 0.01 to 0.36), male gender (OR: 0.35; CI: 0.16 to 0.76), GCS 13 to 15 (OR: 0.28; CI 0.10 to 0.74), high-energy trauma (OR: 9.42; CI 2.15 to 41.20), major extracranial injury (OR: 7.92; CI 2.57 to 24.41), EMS physician telephone consultation (OR: 6.02; CI 2.51 to 14.11) or presence on scene (OR: 8.63; CI 3.50 to 21.26) and incident at a public place outside (OR: 3.05; CI: 1.34 to 6.4) and inside (OR: 2.92; CI: 1.07 to 8.01). Median time delay to trauma center admission was 1:07 hours (IQR: 0.52 to 1:28) for directly transported patients and 4:06 (IQR: 2.54 to 5:43) for those indirectly transported (*P <*0.001). There was a clear trend towards poorer neurological outcome for patients with delayed trauma center admission in univariate analysis (*P *= 0.001).

## Conclusion

Heavily alcohol intoxicated TBI patients are commonly initially transported to a non-neurosurgical trauma center and this may worsen 6-month neurological outcome.
